# (*E*)-4-Methyl-*N*-(2,3,4-trimeth­oxy-6-methyl­benzyl­idene)aniline

**DOI:** 10.1107/S1600536809004115

**Published:** 2009-02-11

**Authors:** Cheng-Yun Wang

**Affiliations:** a Department of Chemistry and Chemical Engineering, Weifang University, Weifang 261061, People’s Republic of China

## Abstract

In the title mol­ecule, C_18_H_21_NO_3_, the dihedral angle between the two benzene rings is 42.2 (2)° and it adopts a *trans* configuration with respect to the central C=N bond.

## Related literature

For the structure of the related compound (*E*)-*N*-(2,3,4-trimeth­oxy-6-methyl­benzyl­idene)naphthalen-1-amine, see: Wang (2009 ▶).
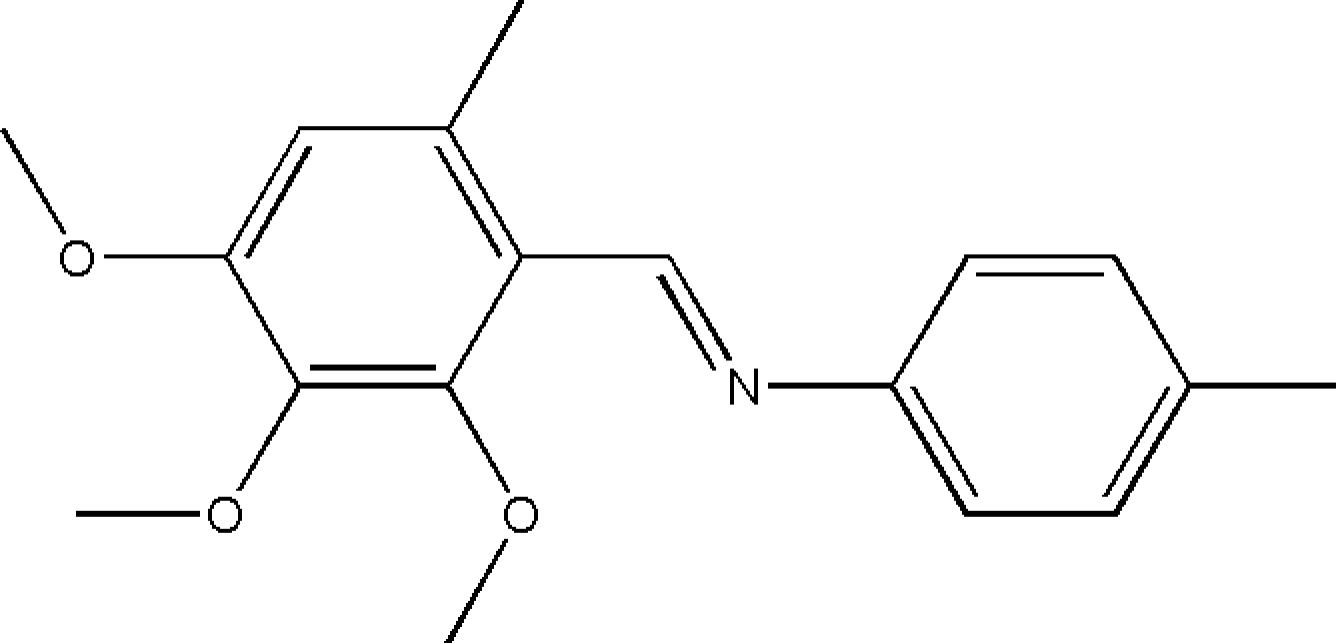


## Experimental

### 

#### Crystal data

C_18_H_21_NO_3_
*M* *_r_* = 299.36
Monoclinic, 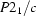

*a* = 7.7239 (9) Å
*b* = 27.287 (2) Å
*c* = 8.4128 (11) Å
β = 111.529 (2)°
*V* = 1649.4 (3) Å^3^
*Z* = 4
Mo *K*α radiation
μ = 0.08 mm^−1^
*T* = 298 (2) K
0.45 × 0.43 × 0.40 mm

#### Data collection

Bruker SMART CCD diffractometer
Absorption correction: multi-scan (*SADABS*; Sheldrick, 1996 ▶) *T*_min_ = 0.964, *T*_max_ = 0.968
8258 measured reflections
2899 independent reflections
1475 reflections with *I* > 2σ(*I*)
*R*_int_ = 0.055

#### Refinement

*R*[*F* ^2^ > 2σ(*F* ^2^)] = 0.053
*wR*(*F* ^2^) = 0.150
*S* = 1.02
2899 reflections
199 parameters
H-atom parameters constrained
Δρ_max_ = 0.19 e Å^−3^
Δρ_min_ = −0.18 e Å^−3^

### 

Data collection: *SMART* (Bruker, 1997 ▶); cell refinement: *SAINT* (Bruker, 1997 ▶); data reduction: *SAINT*; program(s) used to solve structure: *SHELXS97* (Sheldrick, 2008 ▶); program(s) used to refine structure: *SHELXL97* (Sheldrick, 2008 ▶); molecular graphics: *SHELXTL* (Sheldrick, 2008 ▶); software used to prepare material for publication: *SHELXTL*.

## Supplementary Material

Crystal structure: contains datablocks global, I. DOI: 10.1107/S1600536809004115/lh2770sup1.cif

Structure factors: contains datablocks I. DOI: 10.1107/S1600536809004115/lh2770Isup2.hkl

Additional supplementary materials:  crystallographic information; 3D view; checkCIF report

## Figures and Tables

**Fig. 1.**
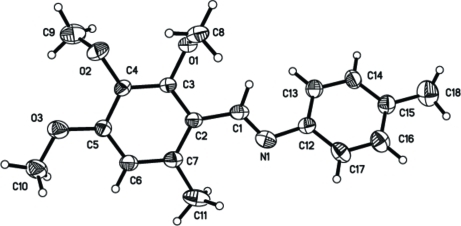
The molecular structure of (I), drawn with 30% probability ellipsoids.

**Fig. 2.**
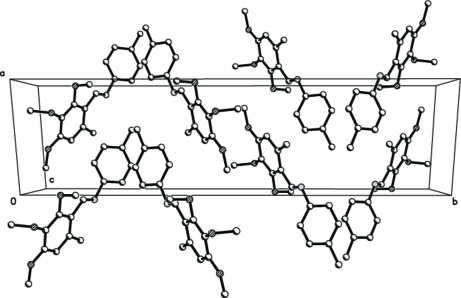
Part of the crystal structure of (I).

## supplementary crystallographic information

### Comment

The preparation, properties and applications of Schiff bases are important in the development of coordination chemistry. In this paper, the structure of the title compound, (I), is reported. The molecular structure of (I) is illustrated in Fig. 1. The bond lengths and angles of the title compound agree with those in the related compound (*E*)-*N*-(2,3,4-trimethoxy-6-methylbenzylidene)naphthalen-1-amine (Wang, 2009), as representative example. The dihedral angle between between the two phenyl rings is 137.8 (2) °. The molecule adopts a *trans* configuration about the central C=N functional bond. In the crystal structure, molecules pack in a 'herring-bone' fashion along the b axis direction (see fig. 2).

### Experimental

A mixture of *p*-toluidine (0.535 g, 5 mmol) and 2,3,4-trimethoxy-6-methylbenzaldehyde (1.04 g, 5 mmol) in ethyl alcohol (20 ml) was stirred magnetically for 2 h at reflux temperature. After cooling the precipitate was filtered and dried. The crude product of 20 mg was dissolved in a 20 ml of ethylalcohol by heating on a magnetic stirrer. The solution was filtered to remove impurities, and then left to crystallize at room temperature. After a week single crystals suitable for the X-ray crystal structure determination were obtained.

### Refinement

The H atoms were positioned geometrically (C—H = 0.93–0.96 Å) and refined as riding with *U*_iso_(H) = 1.2*U*_eq_(C) or 1.5 *U*_eq_(methyl C).

### Crystal data

**Table:**

C_18_H_21_NO_3_	*F*(000) = 640
*M**_r_* = 299.36	*D*_x_ = 1.206 Mg m^−^^3^
Monoclinic, *P*2_1_/*c*	Mo *K*α radiation, λ = 0.71073 Å
Hall symbol: -P 2ybc	Cell parameters from 1422 reflections
*a* = 7.7239 (9) Å	θ = 2.7–20.0°
*b* = 27.287 (2) Å	µ = 0.08 mm^−^^1^
*c* = 8.4128 (11) Å	*T* = 298 K
β = 111.529 (2)°	Block, brown
*V* = 1649.4 (3) Å^3^	0.45 × 0.43 × 0.40 mm
*Z* = 4	

### Data collection

**Table:**

Bruker SMART CCD diffractometer	2899 independent reflections
Radiation source: fine-focus sealed tube	1475 reflections with *I* > 2σ(*I*)
graphite	*R*_int_ = 0.055
φ and ω scans	θ_max_ = 25.0°, θ_min_ = 1.5°
Absorption correction: multi-scan (*SADABS*; Sheldrick, 1996)	*h* = −9→9
*T*_min_ = 0.964, *T*_max_ = 0.968	*k* = −30→32
8258 measured reflections	*l* = −9→5

### Refinement

**Table:**

Refinement on *F*^2^	Primary atom site location: structure-invariant direct methods
Least-squares matrix: full	Secondary atom site location: difference Fourier map
*R*[*F*^2^ > 2σ(*F*^2^)] = 0.053	Hydrogen site location: inferred from neighbouring sites
*wR*(*F*^2^) = 0.150	H-atom parameters constrained
*S* = 1.02	*w* = 1/[σ^2^(*F*_o_^2^) + (0.0477*P*)^2^ + 0.6503*P*] where *P* = (*F*_o_^2^ + 2*F*_c_^2^)/3
2899 reflections	(Δ/σ)_max_ < 0.001
199 parameters	Δρ_max_ = 0.19 e Å^−^^3^
0 restraints	Δρ_min_ = −0.18 e Å^−^^3^

### Special details

**Table:**

Geometry. All e.s.d.'s (except the e.s.d. in the dihedral angle between two l.s. planes) are estimated using the full covariance matrix. The cell e.s.d.'s are taken into account individually in the estimation of e.s.d.'s in distances, angles and torsion angles; correlations between e.s.d.'s in cell parameters are only used when they are defined by crystal symmetry. An approximate (isotropic) treatment of cell e.s.d.'s is used for estimating e.s.d.'s involving l.s. planes.
Refinement. Refinement of *F*^2^ against ALL reflections. The weighted *R*-factor *wR* and goodness of fit *S* are based on *F*^2^, conventional *R*-factors *R* are based on *F*, with *F* set to zero for negative *F*^2^. The threshold expression of *F*^2^ > σ(*F*^2^) is used only for calculating *R*-factors(gt) *etc*. and is not relevant to the choice of reflections for refinement. *R*-factors based on *F*^2^ are statistically about twice as large as those based on *F*, and *R*- factors based on ALL data will be even larger.

### Fractional atomic coordinates and isotropic or equivalent isotropic displacement parameters (Å^2^)

**Table:**

	*x*	*y*	*z*	*U*_iso_*/*U*_eq_	
N1	0.9325 (3)	0.15463 (10)	0.3804 (4)	0.0607 (7)	
O1	0.9997 (3)	0.09590 (7)	−0.0287 (2)	0.0536 (6)	
O2	0.7379 (3)	0.04533 (8)	−0.2839 (3)	0.0628 (6)	
O3	0.3972 (3)	0.02973 (8)	−0.2724 (3)	0.0640 (6)	
C1	0.9412 (4)	0.13395 (11)	0.2492 (4)	0.0520 (8)	
H1	1.0549	0.1351	0.2348	0.062*	
C2	0.7898 (4)	0.10855 (10)	0.1187 (4)	0.0448 (7)	
C3	0.8252 (4)	0.08912 (10)	−0.0207 (4)	0.0451 (7)	
C4	0.6955 (4)	0.06230 (10)	−0.1484 (4)	0.0466 (7)	
C5	0.5201 (4)	0.05545 (10)	−0.1404 (4)	0.0481 (8)	
C6	0.4808 (4)	0.07464 (11)	−0.0057 (4)	0.0506 (8)	
H6	0.3632	0.0698	−0.0019	0.061*	
C7	0.6114 (4)	0.10096 (10)	0.1238 (4)	0.0512 (8)	
C8	1.0063 (5)	0.13609 (12)	−0.1356 (5)	0.0742 (11)	
H8A	0.9214	0.1302	−0.2502	0.111*	
H8B	1.1303	0.1393	−0.1350	0.111*	
H8C	0.9717	0.1657	−0.0933	0.111*	
C9	0.7579 (5)	−0.00633 (14)	−0.2860 (5)	0.0790 (11)	
H9A	0.8493	−0.0169	−0.1793	0.119*	
H9B	0.7973	−0.0154	−0.3777	0.119*	
H9C	0.6408	−0.0217	−0.3023	0.119*	
C10	0.2178 (4)	0.01986 (14)	−0.2686 (4)	0.0737 (11)	
H10A	0.2309	0.0048	−0.1617	0.111*	
H10B	0.1515	−0.0018	−0.3607	0.111*	
H10C	0.1499	0.0500	−0.2808	0.111*	
C11	0.5545 (5)	0.11989 (13)	0.2664 (5)	0.0774 (11)	
H11A	0.4251	0.1131	0.2402	0.116*	
H11B	0.5749	0.1546	0.2779	0.116*	
H11C	0.6275	0.1040	0.3715	0.116*	
C12	1.0977 (4)	0.17428 (11)	0.5029 (4)	0.0501 (8)	
C13	1.2692 (4)	0.15170 (11)	0.5499 (4)	0.0566 (9)	
H13	1.2816	0.1228	0.4965	0.068*	
C14	1.4218 (4)	0.17184 (12)	0.6755 (4)	0.0623 (9)	
H14	1.5363	0.1562	0.7045	0.075*	
C15	1.4108 (4)	0.21420 (12)	0.7595 (4)	0.0596 (9)	
C16	1.2394 (5)	0.23596 (12)	0.7127 (4)	0.0678 (10)	
H16	1.2272	0.2647	0.7668	0.081*	
C17	1.0854 (5)	0.21645 (12)	0.5881 (4)	0.0653 (10)	
H17	0.9708	0.2319	0.5607	0.078*	
C18	1.5798 (5)	0.23466 (14)	0.8988 (5)	0.0950 (13)	
H18A	1.6665	0.2465	0.8501	0.143*	
H18B	1.6375	0.2094	0.9806	0.143*	
H18C	1.5431	0.2612	0.9546	0.143*	

### Atomic displacement parameters (Å^2^)

**Table:**

	*U*^11^	*U*^22^	*U*^33^	*U*^12^	*U*^13^	*U*^23^
N1	0.0504 (16)	0.0693 (19)	0.0638 (19)	−0.0076 (14)	0.0229 (15)	−0.0111 (15)
O1	0.0471 (12)	0.0616 (14)	0.0591 (14)	−0.0011 (10)	0.0279 (10)	0.0078 (11)
O2	0.0767 (15)	0.0720 (16)	0.0501 (14)	−0.0086 (12)	0.0355 (12)	−0.0009 (11)
O3	0.0516 (13)	0.0881 (17)	0.0500 (14)	−0.0170 (12)	0.0159 (11)	−0.0071 (12)
C1	0.0486 (19)	0.0546 (19)	0.061 (2)	−0.0062 (15)	0.0293 (17)	0.0001 (17)
C2	0.0424 (17)	0.0433 (17)	0.0525 (19)	−0.0031 (14)	0.0220 (15)	−0.0009 (14)
C3	0.0414 (17)	0.0468 (18)	0.0517 (19)	−0.0009 (14)	0.0225 (15)	0.0067 (15)
C4	0.0497 (19)	0.0529 (19)	0.0403 (18)	−0.0016 (15)	0.0200 (15)	0.0060 (15)
C5	0.0437 (18)	0.0512 (19)	0.0470 (19)	−0.0039 (15)	0.0140 (15)	0.0036 (15)
C6	0.0449 (18)	0.0546 (19)	0.056 (2)	−0.0047 (15)	0.0224 (16)	0.0009 (16)
C7	0.0529 (19)	0.0495 (19)	0.059 (2)	−0.0036 (15)	0.0292 (17)	−0.0030 (16)
C8	0.068 (2)	0.075 (2)	0.095 (3)	−0.0027 (19)	0.048 (2)	0.023 (2)
C9	0.090 (3)	0.081 (3)	0.082 (3)	−0.008 (2)	0.051 (2)	−0.016 (2)
C10	0.053 (2)	0.099 (3)	0.063 (2)	−0.0190 (19)	0.0128 (18)	−0.003 (2)
C11	0.067 (2)	0.088 (3)	0.095 (3)	−0.021 (2)	0.052 (2)	−0.033 (2)
C12	0.0501 (19)	0.0525 (19)	0.0501 (19)	−0.0024 (16)	0.0212 (16)	0.0005 (16)
C13	0.062 (2)	0.0442 (19)	0.067 (2)	0.0038 (17)	0.0269 (19)	−0.0008 (16)
C14	0.049 (2)	0.059 (2)	0.072 (2)	0.0071 (17)	0.0150 (18)	0.0057 (19)
C15	0.059 (2)	0.051 (2)	0.061 (2)	−0.0044 (17)	0.0131 (18)	−0.0014 (17)
C16	0.068 (2)	0.056 (2)	0.075 (2)	0.0058 (19)	0.021 (2)	−0.0140 (18)
C17	0.052 (2)	0.066 (2)	0.076 (3)	0.0088 (17)	0.0201 (19)	−0.0124 (19)
C18	0.077 (3)	0.087 (3)	0.091 (3)	−0.009 (2)	−0.005 (2)	−0.012 (2)

### Geometric parameters (Å, °)

**Table:**

N1—C1	1.263 (3)	C9—H9B	0.9600
N1—C12	1.419 (4)	C9—H9C	0.9600
O1—C3	1.386 (3)	C10—H10A	0.9600
O1—C8	1.431 (3)	C10—H10B	0.9600
O2—C4	1.377 (3)	C10—H10C	0.9600
O2—C9	1.419 (4)	C11—H11A	0.9600
O3—C5	1.361 (3)	C11—H11B	0.9600
O3—C10	1.423 (3)	C11—H11C	0.9600
C1—C2	1.453 (4)	C12—C17	1.377 (4)
C1—H1	0.9300	C12—C13	1.380 (4)
C2—C3	1.402 (4)	C13—C14	1.376 (4)
C2—C7	1.409 (4)	C13—H13	0.9300
C3—C4	1.380 (4)	C14—C15	1.373 (4)
C4—C5	1.393 (4)	C14—H14	0.9300
C5—C6	1.380 (4)	C15—C16	1.370 (4)
C6—C7	1.384 (4)	C15—C18	1.505 (4)
C6—H6	0.9300	C16—C17	1.372 (4)
C7—C11	1.512 (4)	C16—H16	0.9300
C8—H8A	0.9600	C17—H17	0.9300
C8—H8B	0.9600	C18—H18A	0.9600
C8—H8C	0.9600	C18—H18B	0.9600
C9—H9A	0.9600	C18—H18C	0.9600
			
C1—N1—C12	118.8 (3)	O3—C10—H10A	109.5
C3—O1—C8	113.1 (2)	O3—C10—H10B	109.5
C4—O2—C9	113.8 (2)	H10A—C10—H10B	109.5
C5—O3—C10	118.2 (2)	O3—C10—H10C	109.5
N1—C1—C2	125.9 (3)	H10A—C10—H10C	109.5
N1—C1—H1	117.0	H10B—C10—H10C	109.5
C2—C1—H1	117.0	C7—C11—H11A	109.5
C3—C2—C7	117.6 (3)	C7—C11—H11B	109.5
C3—C2—C1	117.5 (3)	H11A—C11—H11B	109.5
C7—C2—C1	124.9 (3)	C7—C11—H11C	109.5
C4—C3—O1	118.3 (3)	H11A—C11—H11C	109.5
C4—C3—C2	122.9 (3)	H11B—C11—H11C	109.5
O1—C3—C2	118.8 (3)	C17—C12—C13	117.8 (3)
O2—C4—C3	119.9 (3)	C17—C12—N1	118.6 (3)
O2—C4—C5	121.7 (3)	C13—C12—N1	123.5 (3)
C3—C4—C5	118.3 (3)	C14—C13—C12	120.2 (3)
O3—C5—C6	124.6 (3)	C14—C13—H13	119.9
O3—C5—C4	115.4 (3)	C12—C13—H13	119.9
C6—C5—C4	120.0 (3)	C15—C14—C13	122.3 (3)
C5—C6—C7	121.8 (3)	C15—C14—H14	118.9
C5—C6—H6	119.1	C13—C14—H14	118.9
C7—C6—H6	119.1	C16—C15—C14	116.9 (3)
C6—C7—C2	119.4 (3)	C16—C15—C18	122.2 (3)
C6—C7—C11	117.5 (3)	C14—C15—C18	120.9 (3)
C2—C7—C11	123.1 (3)	C15—C16—C17	121.8 (3)
O1—C8—H8A	109.5	C15—C16—H16	119.1
O1—C8—H8B	109.5	C17—C16—H16	119.1
H8A—C8—H8B	109.5	C16—C17—C12	121.0 (3)
O1—C8—H8C	109.5	C16—C17—H17	119.5
H8A—C8—H8C	109.5	C12—C17—H17	119.5
H8B—C8—H8C	109.5	C15—C18—H18A	109.5
O2—C9—H9A	109.5	C15—C18—H18B	109.5
O2—C9—H9B	109.5	H18A—C18—H18B	109.5
H9A—C9—H9B	109.5	C15—C18—H18C	109.5
O2—C9—H9C	109.5	H18A—C18—H18C	109.5
H9A—C9—H9C	109.5	H18B—C18—H18C	109.5
H9B—C9—H9C	109.5		
			
C12—N1—C1—C2	174.7 (3)	O3—C5—C6—C7	−179.3 (3)
N1—C1—C2—C3	178.1 (3)	C4—C5—C6—C7	0.1 (4)
N1—C1—C2—C7	−3.5 (5)	C5—C6—C7—C2	0.1 (4)
C8—O1—C3—C4	83.9 (3)	C5—C6—C7—C11	−179.3 (3)
C8—O1—C3—C2	−97.8 (3)	C3—C2—C7—C6	0.7 (4)
C7—C2—C3—C4	−1.6 (4)	C1—C2—C7—C6	−177.7 (3)
C1—C2—C3—C4	176.9 (3)	C3—C2—C7—C11	−180.0 (3)
C7—C2—C3—O1	−179.8 (2)	C1—C2—C7—C11	1.6 (5)
C1—C2—C3—O1	−1.3 (4)	C1—N1—C12—C17	145.4 (3)
C9—O2—C4—C3	110.9 (3)	C1—N1—C12—C13	−39.0 (4)
C9—O2—C4—C5	−72.6 (3)	C17—C12—C13—C14	−1.3 (5)
O1—C3—C4—O2	−3.4 (4)	N1—C12—C13—C14	−177.0 (3)
C2—C3—C4—O2	178.4 (3)	C12—C13—C14—C15	0.6 (5)
O1—C3—C4—C5	180.0 (2)	C13—C14—C15—C16	0.0 (5)
C2—C3—C4—C5	1.8 (4)	C13—C14—C15—C18	178.7 (3)
C10—O3—C5—C6	−2.9 (4)	C14—C15—C16—C17	0.2 (5)
C10—O3—C5—C4	177.7 (3)	C18—C15—C16—C17	−178.5 (3)
O2—C4—C5—O3	1.9 (4)	C15—C16—C17—C12	−0.9 (5)
C3—C4—C5—O3	178.4 (2)	C13—C12—C17—C16	1.5 (5)
O2—C4—C5—C6	−177.5 (3)	N1—C12—C17—C16	177.4 (3)
C3—C4—C5—C6	−1.0 (4)		
